# Progress in Surgery

**Published:** 1899-04-01

**Authors:** 


### Progress in Surgery.

### GENERAL SURGERY.

*(Continued from page 437, Vol. XXV.)*

**Fractures and Dislocations.**—From a careful study of the urine in a number of fracture cases, Dr. J. L. Howell[10] has gleaned a few conclusions which he says are of practical value to the surgeon in the care and treatment of such cases. If, on examination of the urine, phosphaturia is marked, it would be unwise to allow too early use of the part, and more rest is indicated. In such cases the administration of the hypophosphites of lime, sodium, and potassium, to aid in the formation of bone tissue would be advisable. The results of an examination of the urine in 12 cases of fracture gave the following conclusions:—(1) That during the course of the reparative process after a fracture there is an increased elimination of phosphates amounting to a decided phosphaturia; (2) that there is a large relative increase of the earthy salts in comparison with the alkaline; (3) that as the fracture is repaired and the patient becomes in a condition to resume free occupation this phosphaturia is less marked, and the earthy salts resume their normal relation to the alkaline. Dr. E. M. Corner[11] contributes an interesting thesis on the influence of position in fractures and dislocations as shown by a comparison of these injuries in man and animals. He has examined the records of more than 350 cases of injury in animals, but has dealt only with those which occur both in man and animals under conditions that are comparable. Broadly speaking, fractures are of rare occurrence in the horse in comparison with man, and dislocations are still rarer; animals agree with man in so far that they suffer less often from dislocations than fractures. The principal mechanical reasons for the fact that dislocations are less frequent in animals than man are that very strong muscles and tendons surround the joints and that only simple, limited movements allowed in them. He treats of fractures and dislocations in digitigrade and plantigrade animals; of fractures of the forearm, leg, patella, sesamoid bones, humerus, femur; and of dislocations of the patella, shoulder, and hip, and their distinctive mechanics in man and the horse. The ways in which the different segments of the limbs are placed give rise to the principal differences in fractures and dislocations in the two cases. During his recent work with the X rays Dr. S. Leigh[12] has been much assisted with sprains in showing the amount of abnormal mobility permitted by the injured ligaments, while so many cases of supposed sprains turned out to be fractures that he was convinced these small breaks had been very generally overlooked. The practical advantage of demonstrating the existence of such a fracture is to prevent too early use of the injured part, which might result in permanent harm. He gives an instance of fracture of the tibia and one of fracture of the fibula, both of which had been treated as sprains until detected by the X ray examination. In an article upon fractures of the bones of the face, Dr. C. C. Warden[13] points out that reports from the London and Berlin hospitals show that, out of 1,876 fractures of this region, there were 453 of the inferior maxilla, 268 of the nose, and 142 of the superior maxilla and zygoma. He consequently infers that the inferior maxilla is fractured four times more frequently than the superior maxilla and twice as frequently as the nose. Fracture of the nasal bones is always the result of direct violence. Such injuries are most common in males during the third decade of life, and are quite rare in females and children, particularly among the upper classes. The present athletic tendency of females has to some extent brought up the average, and bicycle riding appears to be a fruitful source of such injury. Usually the fracture is double or multiple, and the deformity—depression and flattening of the bridge—quite apparent. After reduction of the fragments sufficient pressure can be brought to bear upon the parts by using an ordinary hairpin, properly bent and adjusted, the free end of which is embedded in a thin layer of cork, which is retained on the upper lip by adhesive plaster. G. C. Davis,[14] after elevation of the bridge by Sir Astley Cooper's director, thrusts a pin from side to side through the nose, and puts a piece of cork on each projecting end of the pin, pushing the pieces together until the bridge is sufficiently elevated. If not remedied at once the deformity does not tend to disappear. In a lecture on fractures and their treatment, Mr. Arbuthnot Lane[15] deals especially with fractures of the first rib, and injuries of the hip-joint. He points out that previous to his paper in the Guy's Hospital Reports of 1883, the surgeon was entirely unaware of the existence of fracture of the first rib alone, quite apart from possessing any knowledge of the mechanics of its production; indeed, he asserted that "fracture of this rib does not occur, because it is sheltered by the clavicle." If sufficient force is applied to the outer end of the clavicle in any direction, from vertically downwards to almost directly backwards, it will transmit that force to some point in the length of the costal arch, and will break the bone at the point of impact. Should the patient after such a fracture continue to use the arm, a large quantity of callus will be thrown out, because of the free movements resulting from the force exerted on the arch by the clavicle during movements of the arm; and if the arch has yielded in its anterior half the constant movement may produce an ununited fracture. He also relates[16] a case in which a mass of callus had formed about what had evidently been a fracture of the rib in the situation of the subclavian groove. The subclavian artery and brachial plexus were displaced forwards. No pulsation could be detected in the vessels of the arm, which was cold and blue. The fingers were flexed and rigid, and movements at the wrist, elbow, and shoulder were very limited. There was very great pain in the whole extremity. Mr. Lane divided the clavicle, and removed the mass of bone, with the result that complete recovery was ensuing. He has come across several other cases of fracture of the first rib, in which there was clear evidence of compression of the superjacent structures by callus, but in none of them was it necessary to interfere surgically. According to Mr. John Poland's recent treatise on "Traumatic Separation of the Epiphyses," Mr. J. Battersby's[17] case of detachment of the epiphysial head of four lumbar ribs, in a lad aged 14, is a unique one. The injury, however, was direct, the patient having been buried beneath a fall of coal from the roof of a mine. The symptoms and physical signs present were those of ruptured kidney. The large retroperitoneal hæmorrhage, found after death, infiltrating the lumbar muscles and fasciæ, was due to laceration of the lumbar and ascending lumbar veins, which latter vein nominally passes upwards in front of the lumbar transverse processes, *i.e.*, in the line of fracture, which also ran across these processes.

**Upper Extremity.**—J. E. Platt[18] gives an elaborate and statistical paper contributory to the surgery of fractures and dislocations of the upper extremity, based upon an analysis of 700 cases observed at the Manchester Royal Infirmary. The records are of cases which came under his notice from May 20th, 1895, to August 9th, 1896. Out of 25 cases of greenstick fracture of the clavicle he finds this injury most frequent between two and eight years of age. Of 102 cases of complete fracture of the clavicle, 31 were in the middle third and 38 at the junction of outer and middle thirds. Comminution of the bone was present in five cases. Four cases of fracture of the acromion (which he believes with Struthers to be more frequent than is usually supposed) are noted, and one of fracture of the surgical neck of the scapula. One hundred and thirteen cases of fracture of the humerus are included, 20 involving the shaft, 33 the upper end, and 60 the lower end. Ten of the upper end were separations of the upper epiphysis. Nine of these were boys and one a girl. Their ages varied from thirteen months to 17 years; the greater number, however, were over 12 years. Displacement was absent in four cases, one of which was probably an incomplete separation. The diaphysis was displaced inwards in two cases, and forward in four. All the patients had subsequently perfect movement in the shoulder-joint. Of the 20 cases of fracture of the shaft there were 17 males and 3 females; 5 were of the upper third, 11 of the middle third, and 4 of the lower third. Dr. H. R. Wharton[19] also records brief notes of four cases of separation of the upper epiphysis of the humerus in which the results of treatment were satisfactory, especially as regards the junction of the shoulder-joint. He does not think that arrest of growth of the limb in length, although a possible sequel of the injury, is a very common one. The cases which he had under observation for several years showed no difference in the length of the limbs. Dr. G. H. Edington[20] has drawn some diagrams of fracture of the upper and lower ends of the humerus, which present many features of interest. In the former there was a complete fracture of the surgical neck, with some displacement outwards and upwards of the upper end of the lower fragment, so that there was impaction to a certain extent into the head of the bone postero-internally. In the latter, taken from a lad aged 17, there had evidently been a fracture of the external condyle and capitellum, with upward and backward displacement, associated with backward and outward dislocation of the forearm bones. Some brief notes on the practical anatomy of the shoulder are given by Dr. T. H. Manley.[21] He rightly states that much has been written about the difficulty of recognising dislocations of the shoulder, but that this controversy can only be settled by an appeal to physiological anatomy. It must be recognised that there is such a state as physiological dislocation, and such a condition as incomplete dislocation, when the articular head of the humerus has separated from the glenoid cavity, but is yet within the capsule. Strictly speaking, the head is somewhat displaced, but there is no true dislocation. Kocher's method of reduction of recent dislocations of the shoulder by manipulation is now extensively employed as being an excellent procedure, though his explanation of its working has been called in question. Dr. David Waterston[22] sets out a reasonable explanation found by frequently testing the method on cadavera and dissected specimens. The well-known movements are (*a*) elbow approximated to side, outward rotation of the arm, held nearly vertically, elbow flexed till arrested; (*b*) elevation of the elbow forwards and inwards; (*c*) rotation inwards. First, as to the rotation movement, as Mr. Caird has shown, an indented fracture on the anatomical neck is invariably present in a typical subcoracoid dislocation. This indent being caught on the anterior lip of the glenoid forms with it a fixed point, about which rotation occurs, so that the head rolls outwards in front of the rent, which is stretched and gapes. The locking is assisted by the tension of the internal rotators, which press the head back and outwards, as they are wound round the shaft. The elbow is then moved forwards, upwards, and towards the middle line, and the head passes backwards, downwards, and outwards, returning to its socket, the fulcrum being the musculo-fibrous band, consisting of the latissimus dorsi, teres major, and subscapularis, and of the lower part of the capsule. Lastly, the inward rotation disengages the indent from the glenoid lip, since the muscles and capsule attached at the neck are so tense that the neck is fixed, and the head must roll back into the socket; it cannot roll inwards again. Dr. Delagenière[23] reports a case of arthrotomy for old dislocation of the shoulder. The injury was of three and a half months duration, and complicated by a fracture of the scapular spine. Making an incision over the spine of the scapula, he found the fracture viciously united, and after sawing through it and smoothing off the projecting part, cut directly down on the humeral head but without effecting reduction of the dislocation. After cleaning out the thickened capsule filling the glenoid cavity the head could be replaced. In 25 days the patient commenced to move his arm, but the result was far from perfect, active movement was limited, and the arm tingled. The late Professor Nasse[24] has recorded a rare case of infra-clavicular dislocation upon which he had operated three weeks after the accident. Through an anterior incision the extensive hæmorrhagic infiltration of the muscles was found to be transformed into hard, fibrous matter in which the humeral head was immured. The glenoid cavity was covered with fibrous tissue. As there was no prospect of a useful joint, resection of the humerus was performed with a successful result. Only four cases of infra-clavicular dislocation have been examined anatomically. Reduction is always difficult, and was especially so in the above case, where the capsule and muscles were pulled off. Mr. J. Poland[25] showed at the British Orthopædic Society a specimen obtained by excision of old subcoracoid dislocation, with fracture of the great tuberosity. There was no broadening of the shoulder such as was described in text-books. Professor Duplay[26] quotes MM. Panas and Vincent in stating that paralytic conditions of the upper extremity are only possible in intra-coracoid dislocations, but disagrees with them in considering that these paralyses are due to compression of the brachial plexus between the humeral head and the first two ribs. He believes that the injury to the shoulder is solely responsible for their occurrence. Mr. W. McAdam Eccles[27] publishes an account of a somewhat rare instance of traumatic separation of the lower epiphysis of the humerus with displacement forwards. A boy, aged 10, fell from a swing four or five weeks previously. The forearm was flexed to an angle of 145 deg. with the arm, and active extension was impossible. Full flexion, however, could be accomplished without discomfort. A distinct dimple was seen at the tip of the olecranon, and on measurement the projecting part of the humerus on the outer side to the radial styloid process showed an increase in the length of the arm. A skiagraph taken by Dr. F. H. Low showed the ossific centres of the lower epiphysis displaced forwards. Many months later the patient had a useful limb with little deformity.

[10] New York Med. Rec., Oct. 15, 1898, p. 550. [11] Lancet, Oct. 1, 1898, p. 859. [12] Charlotte Med. Jour., Sept. 1898, p. 274. [13] New York Med. Rec., July 23, 1898, p. 109. [14] Annals of Surg., Oct., 1898. [15] Clinic. Jour., Dec. 14, 1898, p. 129. [16] Ibid., Dec. 21, 1898, p. 152. [17] Glasgow Med. Jour., Jan., 1899, p. 46; and Brit. Med. Jour., Jan. 21, 1899, p. 150. [18] Med. Chron., Oct., 1898, p. 1; Nov., 1898, p. 81; Dec., 1898, p. 161; Jan., 1899, p. 257. [19] Annals of Surg., Oct., 1898, p. 462. [20] Glasgow Med. Jour., July, 1898. [21] The Times and Register, Oct., 1898, p. 205. [22] Lancet, Sept. 10, 1898, p. 700. [23] The Post-Graduate, Aug., 1898, p. 711. [24] Lancet, Nov. 24, 1898, p. 1233. [25] Brit. Med. Jour., Mar. 4, 1899. [26] Le Semaine Médicale, Avril 6, 1898, p. 145. [27] Lancet, Sept. 10, 1898, p. 688.

